# Using artificial intelligence-enabled electrocardiogram to predict cardiac resynchronization therapy outcomes of left bundle branch area pacing

**DOI:** 10.1093/europace/euae007

**Published:** 2024-01-16

**Authors:** Jingjing Chen, Fatima M Ezzeddine, Xiaoke Liu, Freddy Del-Carpio Munoz, Paul Friedman, Yong-Mei Cha

**Affiliations:** Department of Cardiovascular Medicine, Affiliated Hospital of Guizhou Medical University, Guiyang, China; Department of Cardiovascular Medicine, Mayo Clinic, Rochester, MN, USA; Department of Cardiovascular Medicine, Mayo Clinic, Rochester, MN, USA; Department of Cardiovascular Medicine, Mayo Clinic, LaCrosse, WI, USA; Department of Cardiovascular Medicine, Mayo Clinic, Rochester, MN, USA; Department of Cardiovascular Medicine, Mayo Clinic, Rochester, MN, USA; Department of Cardiovascular Medicine, Mayo Clinic, Rochester, MN, USA

**Keywords:** Artificial intelligence, Cardiac resynchronization therapy, Electrocardiogram, Heart failure, Left bundle branch area pacing

Artificial intelligence (AI)-enabled electrocardiogram (ECG) has proved to be a valuable screening tool for identifying individuals with reduced left ventricular ejection fraction (LVEF).^1^ At Mayo Clinic, we have successfully developed and integrated an AI algorithm into our 12-lead ECG dashboard, facilitating early and non-invasive detection of reduced LVEF.^2–4^ The objective of this report was to apply our recently developed AI-ECG algorithm in patients who underwent left bundle branch area pacing (LBBAP) to predict cardiac resynchronization therapy (CRT) outcomes in patients with heart failure and reduced ejection fraction (LVEF ≤ 50%).

This retrospective study included patients who underwent LBBAP at the Mayo Clinic Enterprise between October 2018 and February 2023. A 3830 Medtronic Select Secure lumenless pacing lead was implanted in the left bundle branch area as described elsewhere.^5^ Details of the AI-ECG assessment of reduced EF model were previously published.^2,4^ The output of the AI-ECG model is a continuous value between 0 and 1 that represents the ‘probability of low EF (ejection fraction)’. Baseline and post-procedure ECGs were reviewed, and the ‘probability of low EF’ was collected. Transthoracic echocardiography (TTE) studies at >3 months follow-up after LBBAP were also reviewed to assess the early predictive value of AI-ECG for CRT outcomes. An absolute increase of LVEF > 5% was considered as CRT response. The protocol was approved by the Institution Review Board of Mayo Clinic. SPSS Statistics version 28 (IBM, USA) was used for analysis, and two-sided *P* < 0.05 was considered significant.

A total of 263 patients underwent LBBAP. Of these, 94 had a LVEF ≤ 50%, while 169 had normal LVEF at baseline. All 94 patients with LVEF ≤ 50% had paired immediate post-procedure AI-ECG and TTE at a median follow-up of 152.5 (92.8, 321.5) days. The ‘probability of low EF’ on post-procedure AI-ECG was modestly associated with echocardiographic CRT non-response (AUC 0.69 [95% CI: 0.58, 0.80], *P* = 0.002) with a sensitivity of 76.3% and a specificity of 58.9%. *Figure 1A* shows a patient with a LVEF of 29% who had a baseline AI-ECG with a high (71.87%) ‘probability of low EF’ and underwent LBBAP. Immediately after the device implant, the AI-ECG indicated a reduction in the ‘probability of low EF’ from 71.78% to 4.94%, as shown in *Figure 1B*. The pre- and post-procedure ‘probabilities of low EF’ are depicted in *Figure 1C*. The follow-up LVEF in this patient improved from 29% to 52%. The real changes in LVEF by TTE in relation to the ‘probability of low EF’ on post-procedure AI-ECG divided into four quartiles are shown in *Figure 1D*. Patients with a ‘probability of low EF’ < 50% immediately after LBBAP had greater echocardiographic LVEF improvement compared to those with a ‘probability of low EF’ ≥ 50% (11.6% ± 8.7% vs. 4.9% ± 7.5%, *P* < 0.001). After adjusting for age and sex, the patients with a ‘probability of low EF’ < 50% had higher CRT response (OR 3.34 [1.41, 7.89], *P* = 0.006). A total of 20 (21%) patients had a significant reduction in the ‘probability of low EF’ by ≥60% post-device implant, 18 (90%) of those had CRT response at follow-up with an average improvement in LVEF of 16.5 ± 8.3% as compared to 6.3 ± 7.5% (*P* < 0.001) in patients who had a reduction in the ‘probability of low EF’ by <60%.

**Figure 1:**
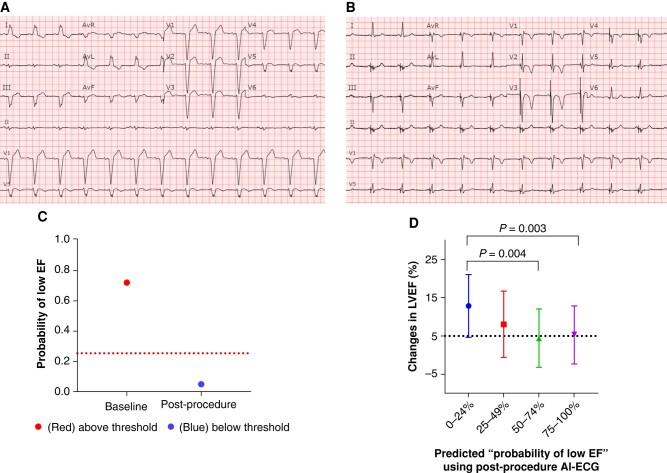
Example of ‘probability of low EF’ derived from AI-ECG and the association with changes in LVEF. Standard 12-lead electrocardiograms were automatically analysed by AI-ECG, providing a predicted ‘probability of low EF’ of 71.87% at baseline (*A*) and of 4.94% post-procedure (*B*) in the same patient. (*C*) The ‘probability of low EF’ decreased from 71.87% at baseline to 4.94% post-procedure, and the LVEF on TTE improved from 29% to 52% at follow-up. (*D*) Changes in LVEF between baseline and follow-up vs. ‘probability of low EF’ on post-procedure AI-ECG. The first quartile group of ‘probability of low EF’ demonstrated a significantly greater improvement in LVEF compared to the third and fourth quartile groups. Other multiple comparisons yielded non-significant *P*-values. AI-ECG, artificial intelligence-enabled electrocardiogram; EF, ejection fraction; LVEF, left ventricular ejection fraction; TTE, transthoracic echocardiography.

The study has demonstrated that the ‘probability of low EF’ derived from AI-ECG can be a potential predictor of CRT outcomes for patients undergoing LBBAP. If it is reproducible in a larger study, AI-ECG may hold the potential to predict CRT response at a specific lead position through AI deep learning of ECG presentations, even in the absence of advanced electrophysiological knowledge and equipment for left bundle branch capture identification. To validate its effectiveness, a prospective clinical study is essential to analyse the variations in the ‘probability of low EF’ during lead implantation before and after left bundle branch capture and the role of these changes in predicting clinical outcomes.

## Data Availability

The data that support the findings of this study are available from the corresponding author, Y.-M.C., upon reasonable request.
